# PainVision-based evaluation of brain potentials: a novel approach for quantitative pain assessment

**DOI:** 10.3389/fbioe.2023.1197070

**Published:** 2023-06-29

**Authors:** Li Chen, Zhen Zhang, Rui Han, Liyuan Du, Zhenxing Li, Shuiping Liu, Dong Huang, Haocheng Zhou

**Affiliations:** ^1^ Department of Pain, The Third Xiangya Hospital and Institute of Pain Medicine, Central South University, Changsha, China; ^2^ Department of Pain, Hunan Prevention and Treatment Institute for Occupational Diseases, Changsha, China; ^3^ Hunan Key Laboratory of Brain Homeostasis, Central South University, Changsha, China

**Keywords:** pain assessment, quantitative sensory testing, PainVision, event-related potential, EEG

## Abstract

**Introduction:** The complex and multidimensional nature of pain poses a major challenge in clinical pain assessments. In this study, we aimed to evaluate a novel approach combining quantitative sensory testing (QST) with event-related potential measurements for assessment of experimental pain in healthy individuals.

**Methods:** QST was performed with a commercial device (PainVision, PS-2100), and numeric rating scale (NRS) scores after exposure to different sensory stimuli were reported by the participants. Resting-state electroencephalography (EEG) was simultaneously performed to capture the cortical responses to peripheral stimulation.

**Results:** Pain scores increased with the intensity of stimuli, with mean NRS scores of 2.7 ± 1.0 after mild stimuli and 5.6 ± 1.0 after moderate stimuli. A reproducible, significant P2-N2 complex was evoked by both mild and moderately painful stimuli, but not by non-painful stimuli. The latency of pain-related potentials was not significantly different between stimuli. The amplitudes of both P2 and N2 components significantly increased when intense nociception was applied, and the increments mainly originated from theta oscillations.

**Conclusion:** The combination of QST with EEG was feasible for subjective and objective pain assessment. Distinct patterns of brain potentials were associated with the phenotype of the peripheral stimuli (e.g., noxious versus. innoxious, high versus. low pain intensity).

## 1 Introduction

Pain assessment is a complex procedure that may occasionally yield significantly variable results across different sessions even when performed by the same assessor. One solution to obtain relatively stable and reproducible data is to apply quantitative sensory testing (QST) for patients with neurologic symptoms or individuals at the risk of developing neurologic disfunction. For assessment of pain perception, multiple tools have been developed to measure the sensory thresholds of touch, vibration, and thermal sensations (Steven et al., 2009). An emerging device capable of assessing pain intensity quantitatively by delivering adjustable electrical stimuli has been recently used to assess neuropathic pain symptoms by comparing heterogeneous sensory perception and ranking pain severity (Yoshida et al., 2015; Yoshida et al., 2019; Saito et al., 2020).

Nevertheless, a major limitation of QST is the subjective nature of the procedure, which requires patient co-operation and is inevitably affected by the patient’s mental condition, education level, motivation, etc. Moreover, the conjunctive usage of behavioral evaluations or questionnaire (Kim et al., 2014) also relies on self-reported results, which are potentially associated with secondary subjective bias. These limitations highlight the need for establishing objective procedures to evaluate sensory-processing functions.

The brain activity information extracted from electroencephalography (EEG) can be used as a biomarker of pain signals, that is, relatively independent of subjective influence. For instance, EEG-based contact heat-evoked potentials can provide objective responses during QST that are not dependent on the subjective characteristics of the tester or examinee (Granovsky et al., 2016; Anders et al., 2022). Thus, the first goal of this study was to combine the QST, in the form of electrical stimuli provided by a commercial PainVision system, with simultaneous EEG recording. Aδ-fibers is mainly responsible for heat pain detection (Nahra and Plaghki, 2003), intraepidermal electrical nociception activates both C- and Aδ-fibers (Inui et al., 2002; Tanaka et al., 2021). The supraspinal mechanism underlying pain signal processing in distinct phenotypes of nociception remains elusive. Consequently, we aimed to investigate the cortical effects of distinct level of electrical stimuli provided by the PainVision-based QST apparatus.

## 2 Methods

### 2.1 Participants

This prospective, observational study was conducted in accordance with the guidelines of the Helsinki Declaration and was approved by the Ethics Committee of The Third Xiangya Hospital, Central South University, China (NO. R22060). Fourteen right-handed Chinese female undergraduates were initially enrolled and consented to undergo the EEG-based QST procedure between November 2022 and December 2022. Two participants were excluded for further analysis due to the low quality of EEG signals. Demographic, behavioral, and neurophysiological data were collected and stored by two independent researchers (ZZ and LD). Written and verbal consent were obtained from all the participants prior to any experiment.

### 2.2 QST procedure

**FIGURE 1:**
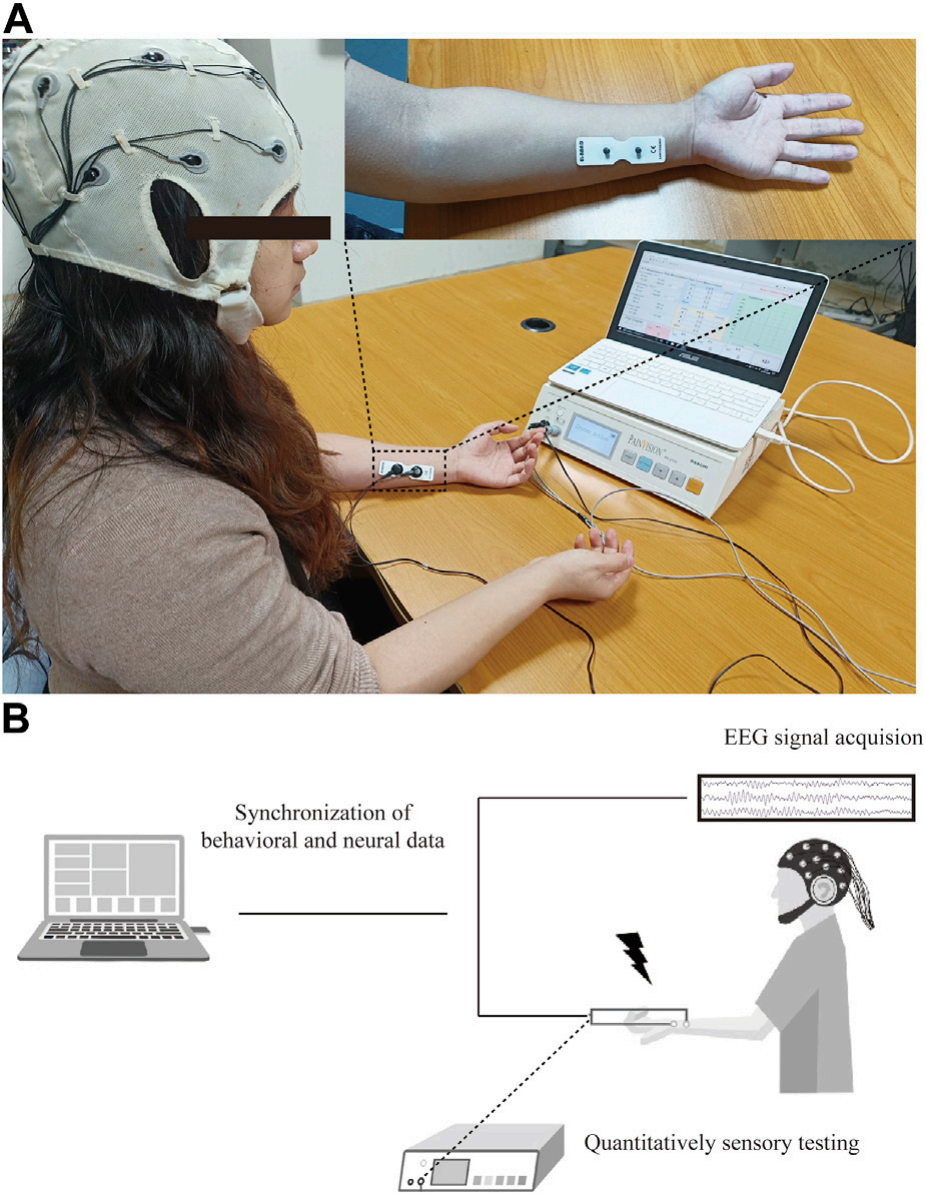
Experimental protocol for PainVision device-based EEG study. **(A)** Set-up of PainVision system, and one 16-channel EEG recording device purchased from OpenBCI company. **(B)** Flow chart of QST-related potentials recording system, EEG data were simultaneously recorded during QST procedure and synchronized for further analysis of ERPs.

QST was performed using a commercial PainVision system (PV, PS-2100, Nipro Co., Osaka, Japan). The experimental device was designed to evaluate sensory or pain thresholds by applying electrical stimuli with different intensities. The detailed QST procedure has been described previously (Kim et al., 2014). Specifically, an expandable electrode (EL-BAND, 200611; Nipro Co., Osaka, Japan) was attached to the skin of the ulnar forearm to deliver electrical stimuli (Figure 1A). The default setting was a stimulation frequency of 50 Hz and 0.3-m pulse width. The output of electrical currents ranged from 0 to 256 μA, and the given intensity of electrical stimuli was applied according to the experimental design and protocol.

For determination of the baseline sensory threshold, each individual was required to press the button of a hand switch twice in one trial. The first press was triggered by the onset of sensation, and consequently indicated the current perception threshold (CPT). The participant was required to press the button the second time if a painful sensation was initially experienced, and this value was then defined as the baseline pain equivalent current (PEC). This protocol for measurement of sensory thresholds was repeated at least three times over a 5-min interval, and the average value was calculated and recorded.

During the EEG recording session, the participants were tested with three levels of stimuli to imitate non-painful (S0), mildly painful (S1), and moderately painful (S2) sensations. The duration of stimuli was set to 1.5, 4, or 5 s in accordance with the clinical guidelines at our center. To measure the stimuli-related potentials, measurements at each intensity were repeated 30 times with a 30-s interval. The stimulus protocol was designed to induce replicable painful sensations while avoiding excessive discomfort to the participants.

Pain severity was further confirmed by assessments using a self-reported numeric rating scale (NRS), in which severity was classified as pain-free (NRS = 0), mild pain (NRS 1–3), moderate pain (NRS 4–6), severe pain (7–9), and worst pain imaginable (NRS 10). Only mild-to-moderate painful sensations were scheduled to prevent overactivation of the peripheral sensory receptors.

### 2.3 Resting-state EEG recording

Resting-state EEG recording was performed as described previously (Zhou H. et al., 2022; Zhang et al., 2022). The participant was seated in a quiet, temperature-controlled, and electrically shielded room, and kept salient and awake with eyes closed during recording session. A commercial 16-channel EEG kit using bio-sensors (Cyton + Daisy, www.OpenBCI.com) was used for acquiring the EEG signal. EEG data recorded at the Cz channel were extracted for event-related potential (ERP) analysis. To guarantee the quality of the signal, the impedance of each recording channel was maintained at approximately 10 kΩ. The sampling rate of the EEG signal recording was set to 125 Hz. The schematic for data processing is shown in Figure 1B.

### 2.3 EEG data processing

EEG data were initially stored in the OpenBCI GUI software and transferred into MATLAB (R2018b, MathWorks, Natick, MA, United States) for further processing. The EEG raw data were preprocessed using the open-source EEGLAB and ERPLAB toolbox (Delorme and MakeigEEGLAB, 2004; Lopez-Calderon and Luck, 2014) by an independent researcher (ZZ). First, all EEG raw traces were manually checked to reject artifacts and malfunctioning channels. The continuous EEG data were then filtered by a band-pass filter (1–30 Hz), and the re-referencing method was used by averaging the values for all scalp channels and then subtracting the resulting signal from each channel. The stimulation events were automatically labeled in the EEG dataset, and the EEG epochs were obtained using the period between −500 and 500 m, in which the 0 point denoted the end of the stimuli. An independent component analysis algorithm was applied for identification and exclusion of artifacts caused by eye blinks, heart beats, and movement. Epochs were also rejected if the amplitudes of the potentials exceeded ± 80 µV. ERPs were averaged across all successful QST trials separately for each stimulus intensity. The P200 (P2) component was measured as the most positive local amplitude between 150 and 250 m post peripheral stimulation, and 250–350 m negative potentials for the later potentials N300 (N2) component respectively.

### 2.4 Statistical analysis

Descriptive analysis was performed to evaluate the demographic features of the participants. Data were presented as mean ± standard deviation. A one-way analysis of variance (ANOVA) with repeated measures using post-hoc Tukey correction was used to compare the NRS scores for different stimuli intensities reported by the participants. The latency and amplitudes of P2, N2, and P2-to-N2 components were compared between mild (S1) and moderate pain stimuli (S2) by using a paired Student’s t-test. One-way ANOVA was performed for event-related spectral perturbation (ERSP) and inter-trial coherence (ITC) analysis, with permutation test and FDR correction across different stimulation outputs. Spearman’s correlation testing was conducted due to non-normal distribution of data. A two-tailed *p*-value less than 0.05 was considered statistically significant for all tests. All statistical analyses were conducted using Prism v8 (GraphPad, San Diego, CA, United States).

## 3 Results

### 3.1 Demographic and behavioral results

Twelve female college students (mean age, 20.4 ± 0.9 years) were enrolled in this study. The electrical outputs corresponding to the CPT and PEC were 10.3 ± 1.9 and 18.0 ± 2.9 µA, respectively. To deliver non-painful stimuli, approximately 30% of the CPT intensity was used for the S0 stimuli (3.8 ± 0.1 µA). The total output in S1 was 33.8 ± 2.8 µA for inducing mild pain and that in S2 was 59.5 ± 3.8 µA for inducing moderate pain. Self-reported NRS scores significantly increased with stimulus intensity, and all participants reported being pain-free (NRS score: 0) during the S0 intervals. The participant characteristics are presented in Table 1.

**TABLE 1:** Demographic and QST data of enrolled participants.

Index	Mean ± sd	Statistics
Age, (years)	20.4 ± 0.9	
BMI, (kg/m^2^)	20.1 ± 2.0	
CPT, (uA)	10.3 ± 1.9	
EPC, (uA)	18.0 ± 2.9	
Stimuli intensity, (uA)		
S0	3.8 ± 0.1	
S1	33.8 ± 2.8	
S2	59.5 ± 3.8	
NRS		
S0	0	
S1	2.7 ± 1.0	
S2	5.6 ± 1.1	0.00****

**** *p*-value <0.0001, N = 12, one-way ANOVA, with repeated measures using post-hoc Tukey correction.

### 3.2 PainVision apparatus-based potentials

**FIGURE 2:**
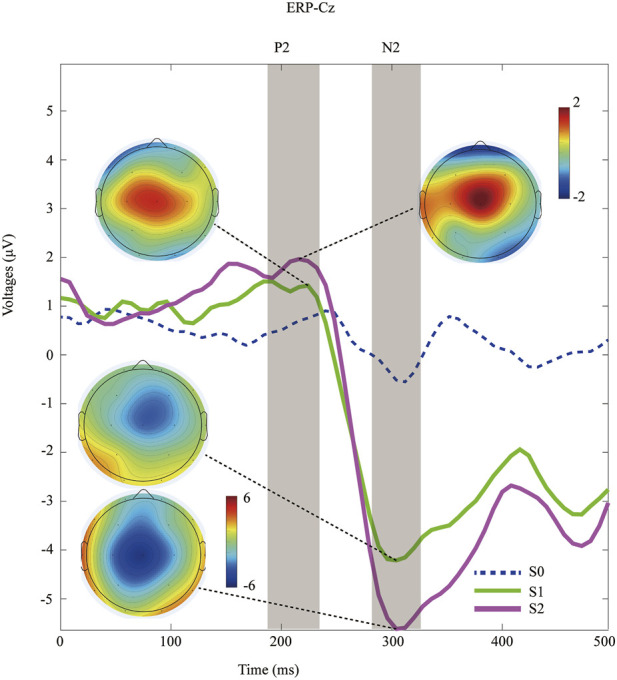
Time-domain data of ERPs recorded at Cz channel during distinct peripheral sensory testing induced by PainVision device. Topography of the P2 and N2 component after nociceptive stimuli (S1 versus. S2).

Peri-stimulus EEG data were recorded at the Cz channel, and the grand-average ERP waves with application of non-nociceptive (S0) or nociceptive stimuli (S1, S2) were subsequently plotted, as shown in Figure 2. The P2 effect was inspected in the present study, and was similar to the window post painful electrical stimuli (Huang et al., 2017). The latency of P2 component was 212.7 ± 24.9 ms after offset of S1 stimuli, and 215.3 ± 23.5 ms in the S2 sessions (*p* = 0.70, paired Student`s t testing). The later components of N2 components were only detected after nociceptive input, but not after control stimuli (blue dashed line in Figure 2). Likewise, we did not find statistically significance in N2 latency between S1 (304.7 ± 24.7 ms) and S2 (309.3 ± 21.7 ms), with a *p*-value of 0.49 calculated with paired Student`s *t* testing.

In contrast, the amplitude of ERPs (P2, N2, and P2-to-N2) was significantly enhanced within a moderate pain session than in those treated with mild pain. Specifically, the amplitude of N2 was 2.3 ± 1.1 μV in the S1-treated sessions, and 3.3 ± 1.6 μV for the S2 respectively (*p* < 0.001, paired Student`s t testing). The P2 amplitudes was 5.0 ± 1.9 μV after S1 input, and 6.9 ± 1.8 μV with the S2 respectively (*p* < 0.001, paired Student`s t testing). Similarly, a significant increasing amplitude of P2-to-N2 was associated with S2 stimuli (10.2 ± 2.8 μV) compared with S1 (7.3 ± 2.4 μV, *p* < 0.001, paired Student`s *t* testing). Detail of ERPs parameter is shown in Table 2.

**TABLE 2:** Comparison of ERPs elicited by mild to moderate pain stimulation.

Index	Mild pain (S1)	Moderate pain (S2)	Statistics
P2 latency (ms)	212.7 ± 24.9	215.3 ± 23.5	0.70
N2 latency (ms)	304.7 ± 24.7	309.3 ± 21.7	0.49
P2 amplitude (uV)	2.3 ± 1.1	3.3 ± 1.6	0.00***
N2 amplitude (uV)	5.0 ± 1.9	6.9 ± 1.8	0.00***
P2-N2 amplitude (uV)	7.3 ± 2.4	10.2 ± 2.8	0.00***

*** *p*-value <0.001, N = 12, measured with paired Student’s *t*-test.

### 3.3 Time-frequency results

**FIGURE 3:**
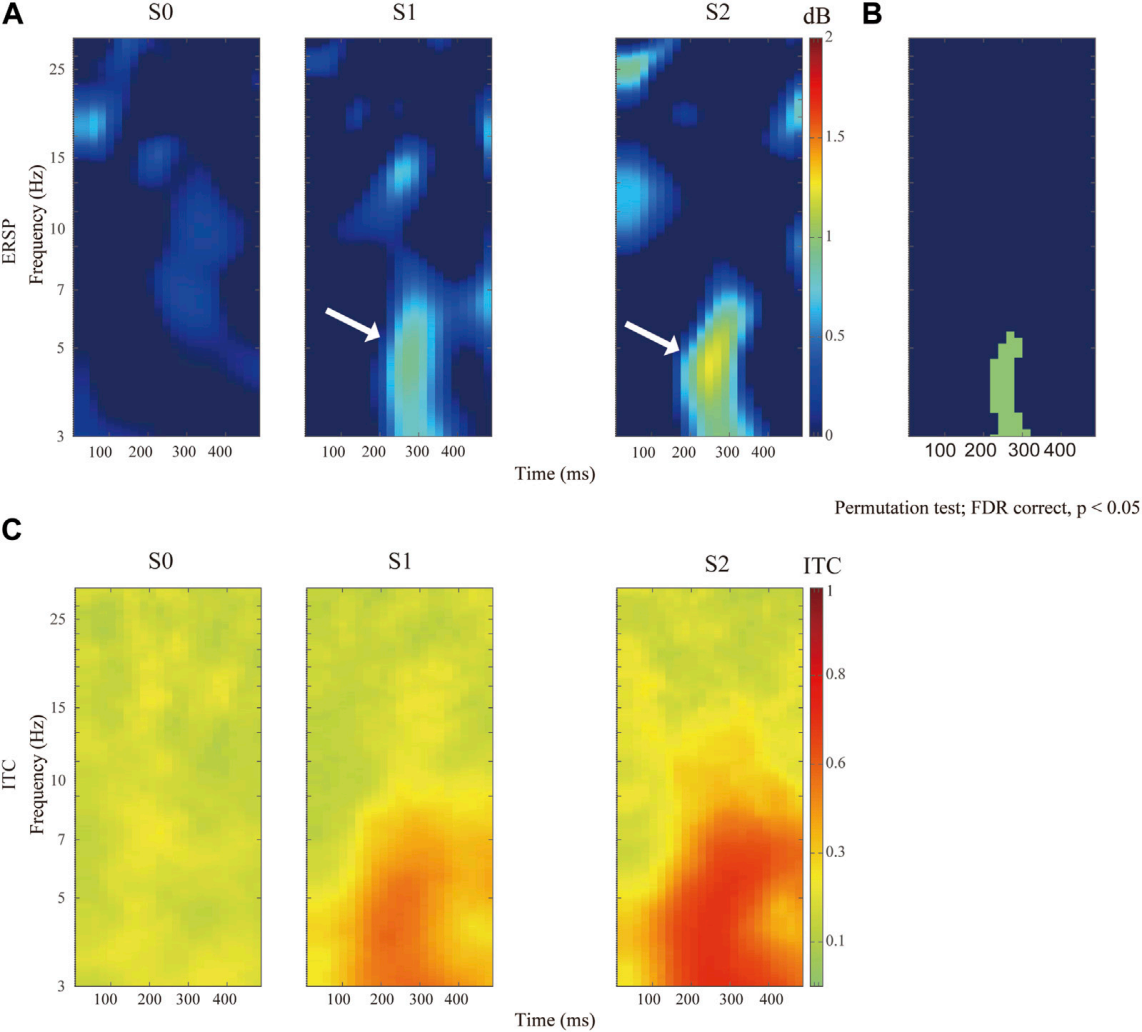
Comparison of distinct stimulation intensity on ERSP recorded at Cz channel. **(A)**The upper panels show the ERSP after application of pain-free stimuli (S0), or painful stimuli for S1 and S2 respectively. **(B)** Comparison of ERSP with non-parametric statistical permutation test, FDR correction across stimulation intensity, *p*-value <0.05 in the green area. **(C)** Identification of P2N2 component with ITC across trials among different stimulation testing.

In general, electrical nociception (S1 and S2) induced a significantly higher P2-to-N2 response (white arrow in Figure 3A). Specifically, the maximal value of ERSP was 0.92 ± 0.76 dB at 4.56 Hz and 272 ms after S1 stimulation, and 1.24 ± 0.87 dB at 4.51 Hz and 248 ms after S2 stimuli respectively (FDR corrected *p*-value = 0.034). A high degree of phase locking was found in painful stimuli (S1 and S2) but not S0, which can be confirmed by the ITC in Figure 3B. Enhanced event-related spectral perturbation (ERSP) was associated with increasing theta (3–7 Hz) activity after painful stimuli in comparison with the control group (Figure 3C).

### 3.4 Relationship between behavioral and cortical response

**FIGURE 4:**
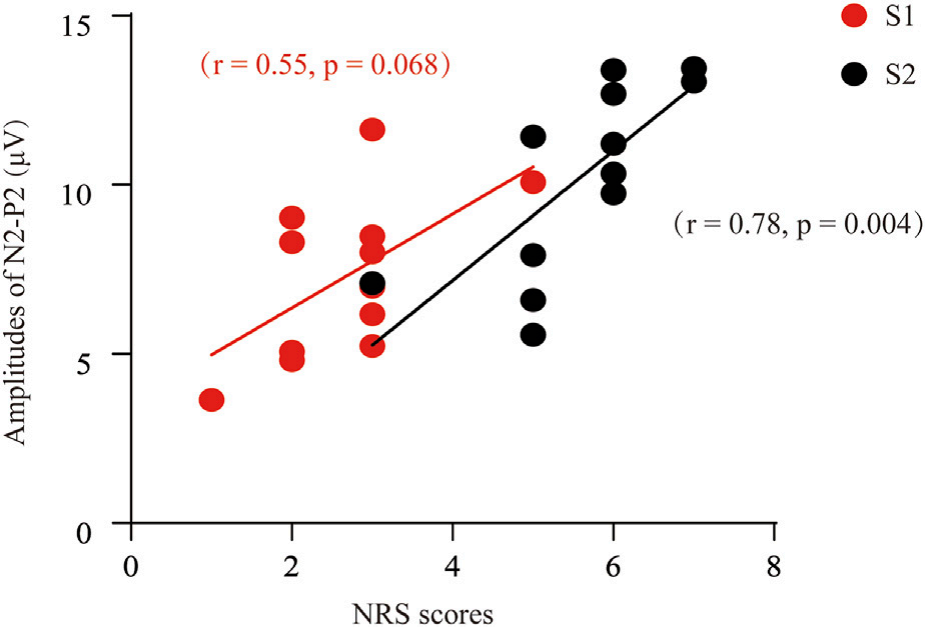
Correlation analysis between the NRS and the P2-to-N2 component of ERPs. A positive and moderate correlation was found between the P2N2 amplitudes and the pain scores after S1 stimuli (*r* = 0.55, *p* = 0.068), or S2 [(*r* = 0.78, *p* = 0.004] respectively.

Next, we determined to test the relationship between the self-reported pain scores and the P2-to-N2 component. In Figure 4, we can find a positive and moderate correlation between the self-reported pain scores (S1) and the amplitude of P2N2 (*r* = 0.55, *p* = 0.068). Likewise, a significant and positive correlation was found between the S2-induced NRS and the amplitude of P2-to-N2 (*r* = 0.78, *p* = 0.004).

## 4 Discussions

The results of QST can be influenced by multiple subjective factors, including mental, physical, cognitive, and motivational status. Therefore, in addition to quantifying the sensory input, objective identification of the evoked responses to peripheral stimuli, which was attempted by EEG measurements of cortical dynamics in this study, is essential. To quantitively capture the characteristics of nociception generated by the commercial PainVision apparatus, we analyzed the ERPs induced by distinct electrical intensities. Our findings may provide supplementary insights regarding pain processing and assessment in both healthy and neuropathic populations.

In this study, QST was conducted with a commercial device (PV, PS-2100, Nipro Co., Osaka, Japan) by delivering percutaneous electrical stimuli at given intensity. In addition to electoral stimulation, several attempts have made to combine ERPs with other phenotype of sensory input (i.e., mechanical, cold, and thermal stimuli) in previous reports (Teel et al., 2022; Anders et al., 2023). We determined to apply PainVision system to achieve a replicable and stable behavioral result by controlling the extract output and duration of peripheral stimuli (Table 1), which is especially critical for data reproducibility in the EEG research. In addition to quantitively monitoring of QST, an obvious implication of this novel device is to imitate the neuropathic features by inducing the electrical sensation (Saito, Odajima, Yokomizo, Tabata, Iida, Ueda, et al). Thus, we think it applicable to conduct this sensory measurement in chronic neuropathic pain cohort in the future study.

The initial goal of QST was to quantify the features of neuropathic pain, with a focus on disruption of sensory impairment (i.e., hyperalgesia and allodynia) (Weaver et al., 2022). Recent data have highlighted the role of QST in the evaluation of sensory dysfunction in chronic pain conditions such as osteoarthritis, fibromyalgia, and chronic low back pain (Brucini et al., 1981; Kosek et al., 1995; Freynhagen et al., 2008). Unlike standardized measurements of the thermal and mechanical threshold (Rolke et al., 2006), the PainVison system quantifies somatosensory perception with electrical impulses, and has been used to assess sensory characteristics in populations of patients experiencing chemotherapy-induced peripheral neuropathy, low back pain, and postherpetic neuralgia (Saito, Odajima, Yokomizo, Tabata, Iida, Ueda, et al.; Wang et al., 2017; Ohtori et al., 2014).

The normal threshold of electrical perception was previously reported to be ≤ 9.4 µA for those aged 20–30 years (Saito, Odajima, Yokomizo, Tabata, Iida, Ueda, et al.), which is consistent with our CPT data. Consequently, a sub-threshold electrical intensity (approximately 30% of the CPT) was determined to induce non-painful stimuli, and the self-reported pain scores (NRS) were used to confirm the actual pain severity subjectively. For delivering mild pain stimulation, the S1 was set to approximately twice the PEC, and the S2 was set to approximately three times the PEC for inducing moderate pain.

One common disadvantage of the self-reported scale and QST mentioned above is that these tools inevitably involve subjective bias, in which the effect of attention is the most studied subject and potentially influences pain perception (Miltner et al., 1989; Le Pera et al., 2002). In this study, we aimed to combine the PainVision system with EEG recordings for objective quantitative pain assessment in healthy volunteers. We think that this approach will be feasible and essential for assessment of specific pain populations in future studies. A potential application of this approach may be in evaluating the cortical response in patients with neuropathic pain and assessing the therapeutic effect after neuromodulation therapy (Zhou H. et al., 2022).

EEG has been widely used in pain research to quantify pain, which is measured with several validated parameters, including ERPs, power spectral density, functional connectivity, and time-frequency domain representations (Tripanpitak et al., 2020). In this study, the distinct somatosensory ERPs elicited by painful and non-painful electrical stimuli were compared. Consistent with the findings of previous studies, the P2 component was significantly identified after nociceptive input, but not after non-painful input, and its amplitude was positively correlated with the stimulation intensity (Miltner et al., 1989). However, our study did not show the appearance of the P3 component instead of N2 onset after the painful stimuli. We assume that in addition to the type of nociception (mechanical, thermal, or electrical) (Granovsky et al., 2016), the stimulation site, duration, and total output of electrical pulses may contribute to the diversity of the main components of ERPs (Miltner et al., 1989; Huang et al., 2017). In addition to electrical stimuli, mechanical and thermal thresholds are more commonly tested in clinical practice to capture the core signs of neuropathic pain syndrome, namely, allodynia and hyperalgesia. Interestingly, mechanical nociception caused by pin-pricks also produced a vertex negative-positive complex, which can be recorded in the Cz channel (Iannetti et al., 2013). Similarly, the N2-to-P2 components induced by infrared laser stimulators have been reported to elicit laser-evoked potentials (Lorenz and Garcia-Larrea, 2003).

An increase in pain perception has been associated with enhancement of theta power in the cortical region (Misra et al., 2017), consistent with our findings for the N2-to-P2 component. Despite the distinct forms of stimuli, the latency of the theta oscillations induced by the PainVision system was close to those elicited by laser stimulation (Shackman et al., 2011; Misra et al., 2017). In addition to pain processing, the theta rhythm has been correlated with memory, arousal, and rapid eye movement (REM) sleep, and is controlled by coordinated propagating activity from different brain regions (Winson, 1978; Zhou Y. et al., 2022). Recently, we have also shown that theta oscillations may contribute to the analgesic effect of electrical nerve stimulation in the management of neuropathic pain (Zhou H. et al., 2022). Given the feasibility of theta power calculation, it may become an objective measure for cognitive assessment (Castro-Meneses et al., 2020) as well as for pain evaluations to overcome the limitations of subjective QST assessments.

In addition to EEG, neuroimaging techniques such as structural magnetic resonance imaging and functional magnetic resonance imaging can also serve as subjective bioinformatics approaches for pain evaluation (van der Miesen et al., 2019), providing neural signals with high temporal resolution. However, the relatively low sampling rate of magnetic resonance imaging may hinder its usage in short-term QST tasks, since the actual timepoint of pain behavior cannot be matched precisely with the imaging data. In contrast, the validity of magnetic resonance imaging in persistent pain conditions has been well established, including its application for capsaicin-induced thermal sensation testing in studies of hyperalgesia and allodynia behavioral phenotypes (Asad et al., 2016). In addition, the structural changes caused by chronic pain conditions can also be identified with task-related or resting-state functional magnetic resonance imaging techniques (Mansour et al., 2013).

The main limitation of this study was the relatively small sample, although we only enrolled young female participants with a similar educational background to avoid potential selection bias. QST for the aged population, especially those with cognitive deficiencies, remains challenging. In addition, one common challenge posing to EEG research is the reproducibility of results, which may be confound by noise or irrelevant signal. Thus, it is necessarily needed to set a standard criterion for data acquisition, pre-processing and analysis of brain signal, as well as in the operation of QST procedure. Finally, future studies should aim to confirm the behavioral and neurophysiological features of patients with specific pain conditions instead of healthy cohorts and employ this novel approach in studies on pain anticipation, relief, and affects.

## 5 Conclusion

The combination of QST using the PainVision system and EEG was a feasible approach. The phenotypes of peripheral stimuli (e.g., noxious versus innoxious stimuli, high versus low pain intensity) may reflect distinct patterns of cortical responses, providing quantitative information about pain processing in the brain.

## Data Availability

The original contributions presented in the study are included in the article/Supplementary Material, further inquiries can be directed to the corresponding author.
